# Supplementary material for: Human-animal relationships and interactions during the Covid-19 lockdown phase in the UK: Investigating links with mental health and loneliness
Source: PLoS One. 2020 Sep 25;15(9):e0239397. doi: 10.1371/journal.pone.0239397 (PMC7518616; doi:10.1371/journal.pone.0239397)
Supplement: S1 Table — (DOCX) [file pone.0239397.s001.docx]

| **S1 Table. Complete participant characteristics.** | | |
| --- | --- | --- |
| **Variable** | | **% (N)** |
| **Gender** | Female | 78.6 (4657) |
|  | Male | 20.6 (1222) |
|  | In another way | 0.6 (36) |
|  | Prefer not to say | 0.2 (11) |
| **Age (years)** | 18 - 24 | 7.1 (420) |
|  | 25 - 34 | 17.5 (1040) |
|  | 35 - 44 | 16.8 (994) |
|  | 45 - 54 | 23.8 (1409) |
|  | 55 - 64 | 22.2 (1313) |
|  | 65 - 70 | 7.1 (418) |
|  | Over 70 | 5.6 (332) |
| **Ethnicity** | White | 96.9 (5742) |
|  | Mixed/multiple ethnic | 1.1 (67) |
|  | Asian/Asian British | 0.5 (32) |
|  | Black/African/Caribbean/Black British | 0.1 (6) |
|  | Chinese | 0.1 (8) |
|  | Arab | 0.1 (4) |
|  | Other ethnic | 0.3 (15) |
|  | Prefer not to say | 0.9 (52) |
| **Education** | GCSE/O-level/CSE | 13.0 (770) |
|  | Vocational qualification | 5.7 (340) |
|  | A-level or equivalent | 16.7 (988) |
|  | Bachelor’s degree or equivalent | 35.0 (2073) |
|  | Masters/PhD or equivalent | 24.0 (1425) |
|  | No formal qualifications | 2.6 (154) |
|  | Other | 3.0 (176) |
| **Employment status** | Self-employed working outside of the home | 2.4 (140) |
|  | Self-employed working from home | 15.2 (903) |
|  | Employed working outside of the home | 29.4 (1745) |
|  | Employed working from home | 6.1 (362) |
|  | Furloughed due to Covid-19 | 11.5 (682) |
|  | Redundant due to Covid-19 | 1.7 (100) |
|  | Unemployed | 4.3 (255) |
|  | Retired | 16.7 (990) |
|  | Student | 3.9 (234) |
|  | Other | 8.8 (515) |
| **Cohabitation** | Live alone | 18.2 (1081) |
|  | With partner/spouse | 61.3 (3630) |
|  | With children < 18 years | 21.1 (1250) |
|  | With adults 18 – 70 years old | 22.8 (1349) |
|  | With adults > 70 years | 3.1 (184) |
|  | With persons who may be vulnerable to Covid-19 | 9.8 (579) |
| **Housing tenure** | Own house with a mortgage | 39.8 (2360) |
|  | Owned outright | 30.8 (1826) |
|  | Rented from local authority | 5.7 (336) |
|  | Rented from private landlord | 15.0 (888) |
|  | Belongs to housing association | 2.8 (167) |
|  | Other | 5.9 (349) |
| **Companion animal ownership** | Yes | 89.8 (5323) |
|  | No | 10.2 (603) |
| **Covid-19 social isolation status** | Socially isolating | 37.4 (2219) |
|  | Not socially isolating | 62.6 (3707) |
