# Supplementary material for: Human-animal relationships and interactions during the Covid-19 lockdown phase in the UK: Investigating links with mental health and loneliness
Source: PLoS One. 2020 Sep 25;15(9):e0239397. doi: 10.1371/journal.pone.0239397 (PMC7518616; doi:10.1371/journal.pone.0239397)
Supplement: S2 Table — (DOCX) [file pone.0239397.s002.docx]

|  | Animal-ownership  % (N) | Non-animal ownership  % (N) |
| --- | --- | --- |
| UCLA score (before lockdown) |  |  |
| 3 | 43.9 (2174) | 39.5 (202) |
| 4 | 15.4 (763) | 15.7 (80) |
| 5 | 11.2 (556) | 15.7 (80) |
| 6 | 17.8 (879) | 16.2 (83) |
| 7 | 4.4 (217) | 4.9 (25) |
| 8 | 3.5 (171) | 3.9 (20) |
| 9 | 3.8 (187) | 4.1 (21) |
| UCLA score (present) |  |  |
| 3 | 25.0 (1237) | 18.0 (92) |
| 4 | 17.8 (879) | 17.0 (87) |
| 5 | 16.2 (803) | 16.2 (83) |
| 6 | 18.8 (928) | 18.6 (95) |
| 7 | 8.2 (407) | 10.8 (55) |
| 8 | 6.4 (318) | 8.0 (41) |
| 9 | 7.5 (375) | 11.4 (58) |
| MHI-5 score (before lockdown) |  |  |
| 5 - 10 | 0.1 (4) | 0.6 (3) |
| 11 - 15 | 2.8 (137) | 2.1 (11) |
| 16 - 20 | 47.5 (2358) | 49.7 (255) |
| 21 - 25 | 48.7 (2417) | 46.8 (240) |
| 26 - 30 | 0.9 (48) | 0.8 (4) |
| MHI-5 score (present) |  |  |
| 5 - 10 | 0.1 (6) | 0.4 (2) |
| 11 - 15 | 2.9 (146) | 3.5 (18) |
| 16 - 20 | 51.9 (2580) | 57.1 (293) |
| 21 - 25 | 44.3 (2194) | 38.6 (198) |
| 26 - 30 | 0.8 (38) | 0.4 (2) |

**S2 Table.** **Mental health and loneliness scores for animal owners and non-owners, pre- and since lockdown.**
